# Impact of different post-harvest processing methods on the chemical compositions of peony root

**DOI:** 10.1007/s11418-018-1214-x

**Published:** 2018-04-13

**Authors:** Shu Zhu, Aimi Shirakawa, Yanhong Shi, Xiaoli Yu, Takayuki Tamura, Naotoshi Shibahara, Kayo Yoshimatsu, Katsuko Komatsu

**Affiliations:** 1 Division of Pharmacognosy, Department of Medicinal Resources, Institute of Natural Medicine, University of Toyama, 2630 Sugitani, Toyama, 930-0194 Japan; 2 Medicinal Plants Center, Toyama Prefectural Institute for Pharmaceutical Research, Kamiichi-Machi, Nakaniikawa-Gun, Toyama, 930-0412 Japan; 3 Division of Kampo Diagnostics, Institute of Natural Medicine, University of Toyama, 2630 Sugitani, Toyama, 930-0194 Japan; 4 Research Center for Medicinal Plant Resources, National Institutes of Biomedical Innovation, Health and Nutrition, 1-2 Hachimandai, Tsukuba, Ibaraki 305-0843 Japan

**Keywords:** Peony root, Post-harvest processing method, Chemical composition, Paeoniflorin, Pentagalloylglucose

## Abstract

The impact of key processing steps such as boiling, peeling, drying and storing on chemical compositions and morphologic features of the produced peony root was investigated in detail by applying 15 processing methods to fresh roots of *Paeonia lactiflora* and then monitoring contents of eight main components, as well as internal root color. The results showed that low temperature (4 °C) storage of fresh roots for approximately 1 month after harvest resulted in slightly increased and stable content of paeoniflorin, which might be due to suppression of enzymatic degradation. This storage also prevented roots from discoloring, facilitating production of favorable bright color roots. Boiling process triggered decomposition of polygalloylglucoses, thereby leading to a significant increase in contents of pentagalloylglucose and gallic acid. Peeling process resulted in a decrease of albiflorin and catechin contents. As a result, an optimized and practicable processing method ensuring high contents of the main active components in the produced root was developed.

**Electronic supplementary material:**

The online version of this article (10.1007/s11418-018-1214-x) contains supplementary material, which is available to authorized users.

## Introduction

Peony root (Paeoniae Radix) is a frequently used herbal drug included in a number of popular formulas in traditional Chinese medicine (TCM) and Kampo medicine [1, 2], called “Shaoyao” in Chinese and “Shakuyaku” in Japanese. Besides the common uses as an ingredient in TCM and Kampo formulas, peony root has also been used in the form of a tincture for treating dyspepsia and menopause disorder in Europe and the United States, and it is also a popular ingredient of functional foods, dietary supplements and cosmetic products. Phytochemical and pharmacological studies have revealed that peony root contains monoterpenoids, flavonoids, tannins and phenols, which are responsible for a variety of peony root bioactivity such as anti-inflammatory, anti-oxidative, anti-coagulative, sedative, analgesic, anti-allergic and anti-hyperglycemic [3–5]. Monoterpenoids with “cage-like” pinane skeleton are characteristic constituents of peony root, among which paeoniflorin has been used as marker component for its quality control.

In China, there are two types of peony root, white peony root (WPR) and red peony root (RPR), which are used for different therapeutic purposes. WPR is prescribed as the boiled, peeled and dried root of *Paeonia lactiflora* Pallas; RPR is prescribed as the dried root of *P. lactiflora* and *P. veitchii* Lynch in Chinese Pharmacopeia [1]. Our previous study has demonstrated that *P. lactiflora*-derived WPR and RPR are not only geographically isolated, but also genetically separated from each other [6, 7]. In addition, WPR and RPR are produced by different post-processing methods. In Japan, peony root has been included in one third of the Kampo formulas and it is prescribed as the root of *P. lactiflora* with not less than 2.0% of paeoniflorin in Japanese Pharmacopeia [8]. Three types of peony root produced by different post-harvest processing methods are available in Japanese market. Those are “Kiboshi-Shakuyaku” (peeled and dried root), “Shinshaku” (peeled, boiled and dried root) and “Kawatsuki-Shakuyaku” (dried root), of which the first is popular. Most peony root used in Japan are imported from China and are produced in the southern part of China the same as WPR, but by different post-harvest processing methods [9].

**Fig. 1:**
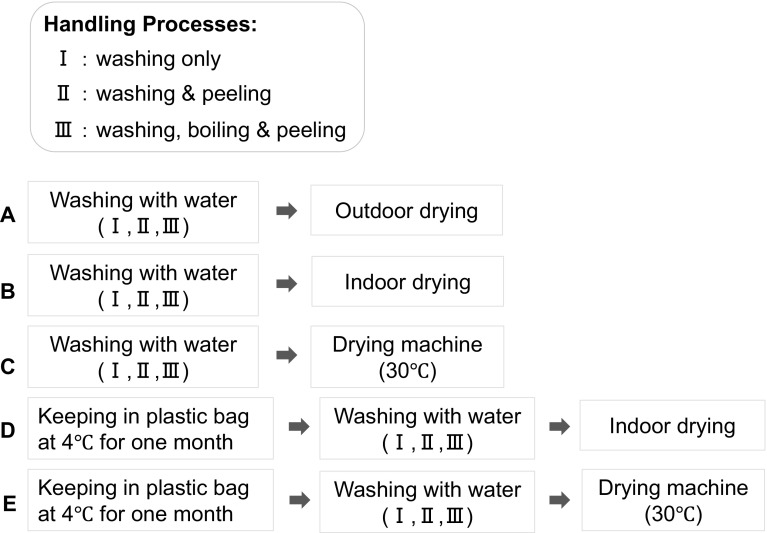
The 15 proposed post-harvest processing methods. (15 groups of fresh roots were processed by these 15 methods, respectively)

**Fig. 2:**
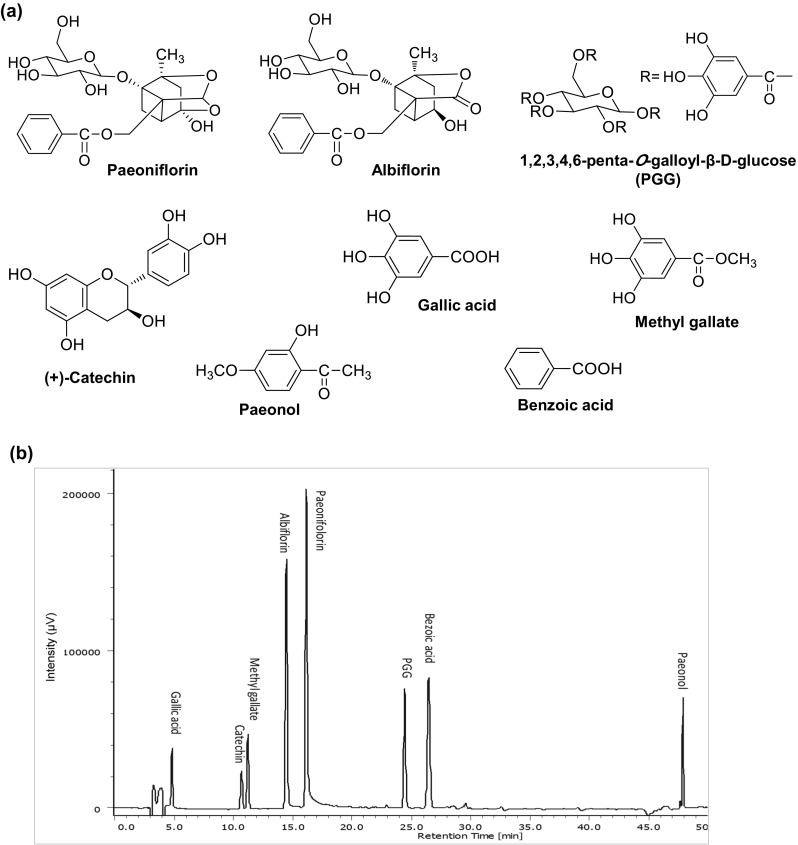
Chemical structures (**a**) and HPLC chromatogram (**b**) of the eight components

Post-harvest processing plays an important role in production of herbal drugs and may affect the organoleptic and chemical properties, as well as clinical efficacy and safety of the produced herbal drugs. A diversity of processing methods have been developed and widely used in TCM, including cleaning, cutting, peeling, boiling, steaming, and stir-frying for the purposes of relieving or decreasing toxicity, changing medicinal properties, improving therapeutic efficiency, etc. [10]. In the case of peony root, post-harvest processing involves boiling, peeling and drying. However, how and to what extent these processing steps affect the chemical composition of the produced roots is not fully understood. In the present work, we proposed 15 processing methods (Fig. 1) to treat the roots of “Bonten”, a cultivar of *P. lactiflora* and then analyzed and compared contents of eight main components (Fig. 2) in the produced roots by HPLC, aiming to figure out the influence of different post-harvest processing methods on the chemical composition and to establish a proper and practicable method for production of brand peony root with superior quality. We also assessed internal color of the produced roots, because plump root with whitish internal color is favored and traditionally believed to be of high quality.

## Materials and methods

### Materials

“Bonten” is a cultivar of *P. lactiflora* developed in Japan for medicinal use and is the botanical source of domestically produced peony root. The 4-year cultivated roots were harvested from the field of the Toyama Prefectural Medicinal Plants Center on Oct. 6, 2013. In order to minimize piece-to-piece variation in chemical constituent contents induced by uneven thickness, the roots with similar diameter were picked up and then randomly divided into 15 groups for the following experiments. Each group with fresh weight of 480–500 g comprised eight individual roots with similar thickness (1.5–2.0 cm in diameter). Two commercial samples of peony root were purchased from Tochimoto Tenkaido Co., Ltd. (TMPW No. 26398) and Uchida Wakanyaku Co., Ltd. (TMPW No. 27890), Japan; the former was produced in Japan and the latter was produced in Anhui prov., China. All the voucher samples were deposited in the Museum of Materia Medica, Institute of Natural Medicine, University of Toyama, Japan (TMPW).

### Fifteen methods for post-harvest processing

Fifteen processing methods, as shown in Fig. 1, were proposed and practiced on the 15 groups of roots, respectively. The proposed methods allow detailed investigation of the impact of processing ways, such as peeling, boiling, drying under different conditions, as well as long-term storage at low-temperature, on the chemical compositions and morphologic features of the produced peony root. Three different handling processes were performed as follows: (I) roots were washed in running water; (II) roots were washed and then peeled; (III) roots were boiled in hot water for 15 min after washing, and then peeled. Three different drying conditions, namely, (A) outdoor (13–31 °C, 53 days), (B) indoor (11–20 °C, 53 days), and (C) kept at 30 °C in a drying machine (38 days) were compared. Thereby, every three groups that were treated by the methods I, II, and III were then dried under the respective three different conditions. In addition, the influence of long-term preservation at low-temperature after harvesting was investigated. Among the 15 groups, six groups (D-I–III and E-I–III groups) were kept in low temperature (4 °C) for approximately 1 month (37 days) before processing, and nine groups (A to C-I–III groups) were immediately processed after harvest.

As for the dried roots, five individuals in each group were used to analyze contents of the eight main components, and to assess the internal root color.

### Quantitative analysis of the eight main constituents by HPLC–DAD

Quantitative analysis of the eight main constituents (Fig. 2a) in the produced peony root was conducted by using the reported method [6]. Of the standards, paeoniflorin, albiflorin, methyl gallate and paeonol were purchased from Wako Pure Chem. Inc. (Osaka, Japan), pentagalloyl glucose (PGG) from Sigma-Aldrich Co. Ltd. (Dorset, UK), (+)-catechin from Nagara Science Inc. (Gifu, Japan), gallic acid and benzoic acid from Nacalai Tesque Inc. (Kyoto, Japan). Reagents for HPLC analysis including acetonitrile, distilled water (both of HPLC grade) and phosphoric acid (analytical grade) were purchased from Wako Pure Chem. Inc. (Japan).

A Jasco HPLC system equipped with a PU-1580 pump, a LC-1580-02 ternary gradient unit, a MD-1510 multiwavelength detector was used. Analysis was carried out using a YMC Pack ODS-AQ (4.6 mm i.d. × 250 mm, 5 μm) with column temperature at 27 °C. Mobile phase consisted of binary eluents of (A) acetonitrile and (B) 0.1% (v/v) phosphoric acid under gradient conditions (0 min, 10% A; 5 min, 15% A; 40 min, 30% A; 45 min, 70% A; 46 min, 80% A; 50 min, 80% A; 55 min, 10% A; 65 min, 10% A. Flow rate was 1.0 ml/min. Detection was performed at wavelength of 232 nm. A baseline separation of the 8 main constituents was achieved (Fig. 2b).

### Preparation of standard and sample solutions

Each standard compound was accurately weighed and dissolved in 75% EtOH to make a stock solution of 1.0 mg/mL. Then a series of standard solutions (200, 100, 20, 10, 2 µg/mL) were prepared through dilution of the stock solution to make calibration curves. Calibration curve of each standard compound was calculated by plotting of peak areas (*y*) against a series of injection amounts (*x*). The calibration equation and correlation coefficient of the eight standard compounds are as follows: gallic acid, *y* = 903638.37*x* + 8265.99 (*R* ^2^ = 0.9999); (+)-catechin, *y* = 2171012.18*x* − 10513.90 (*R* ^2^ = 1.0000); methyl gallate, *y* = 1511887.25*x* − 5964.00 (*R* ^2^ = 0.9999); albiflorin, *y* = 1050787.79x + 11596.65 (*R* ^2^ = 1.0000); paeoniflorin, *y* = 1119831.09*x* − 25391.11 (*R* ^2^ = 0.9993); PGG, *y* = 1502031.75*x* − 6012.82 (*R* ^2^ = 0.9998); benzoic acid, *y* = 4252742.57*x* + 46499.19 (*R* ^2^ = 0.9995); paeonol, *y* = 2874123.93*x* + 15205.35 (*R* ^2^ = 0.9997).

Each root was pulverized and then sieved through 300 µm sieve to obtain homogeneous fine powder from each sample. The fine powder was accurately weighed into 0.3 g and extracted with 75% EtOH (9 mL, 8 mL × 2) by ultra-sonication at room temperature for 30 min, mixed periodically by vortex to obtain full extraction. Then, the supernatant was obtained by centrifugation at 2500 rpm (Kubota 3740, Japan) for 10 min. Supernatants were combined into a 25.0 mL volumetric flask and finally filled with 75% EtOH up to the volume. After filtration through 0.2 µm Millipore filter unit (Advantec, Japan), 20.0 µl of this solution was injected into the HPLC system for quantitative analysis.

### Detecting changes in gallotannins by LC-ESI-IT-TOF–MS analysis

HPLC system (Shimadzu, Kyoto, Japan) consisted of an LC-20AD binary pump, a DGU-14A degasser, a SIL-20AC auto-sampler, a CTO-20AC column oven and a SPD-M20A DAD detector. The mobile phase consisted of (A) acetonitrile containing 0.1% (v/v) formic acid and (B) water containing 0.1% (v/v) formic acid. The sample solutions were analyzed on a YMC Pack ODS-AQ column (2.0 mm i.d. × 150 mm, 3 μm) with a gradient elution system as follows: 00 min, 5% A; 10 min, 10% A; 20 min, 15% A; 25 min, 15% A; 30 min, 20% A; 35 min, 20% A; 45 min, 25% A; 50 min, 40% A. 60 min, 90% A. The flow rate was 0.2 mL/min, and the column temperature was set at 40 °C. A hybrid ion trap time-of-flight (IT-TOF) mass spectrometer (Shimadzu, Tokyo, Japan) equipped with electrospray ionization (ESI) source interface was connected to the Shimadzu HPLC system. TOF mass spectrometer was calibrated using a lock-mass trifluoroacetic acid sodium solution to increase mass accuracy. Negative ion mode was used and the optimized mass parameters were set as follows: Detector voltage, 1.75 kV; spray voltage, − 3.5 kV; nebulizing gas (N_2_) flow, 1.5 L/min; dry gas (N_2_) pressure, 100 kPa; curved desolvation line (CDL) temperature, 200 °C; heat block temperature, 200 °C. Molecular weight acquisition was performed from *m/z* 100 to 2000 for MS and *m/z* 100 to 1000 for MS/MS. The ion accumulation time was set at 30 ms, and the collision energy of collision-induced dissociation (CID) was set as 50%. Data acquisition and processing were performed with the LCMS solution version 3.8 software package (Shimadzu, Tokyo, Japan).

### Assessment of internal root color

After the dried root was cut with hacksaw, color of cross-section was measured by a spectrophotometer NF-333 (Nippon Denshoku Industries Co., Japan). The evaluation system prescribed by the Japanese Industrial Standards (JISZ8729) using L*, a*, b* parameters (CIE L*a*b*) to indicate the color was applied. The L* value represents brightness of color, ranging from black (L* = 0) to white (L* = 1); the a* and b* values describe phase and intensity of color, respectively, of which − a* represents green and + a* represents red, − b* represents blue and + b* represents yellow [11, 12]. Five roots in every group were measured individually and the results were indicated as their average value.

## Results and discussion

Changes in contents of the eight main constituents in the peony roots produced by different post-harvest processing methods.

**Fig. 3:**
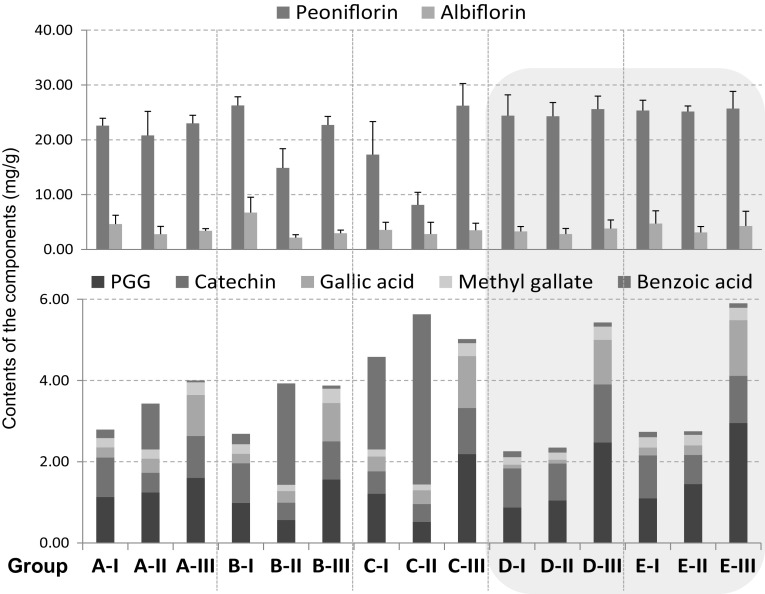
Contents of the seven major components in the roots produced by different post-harvest processing methods (*n* = 5). Average contents and S.D. of paeoniflorin and albiflorin are shown in the upper part and average contents of PGG, gallic acid, catechin, methyl gallate and benzoic acid are shown in the lower part

**Fig. 4:**
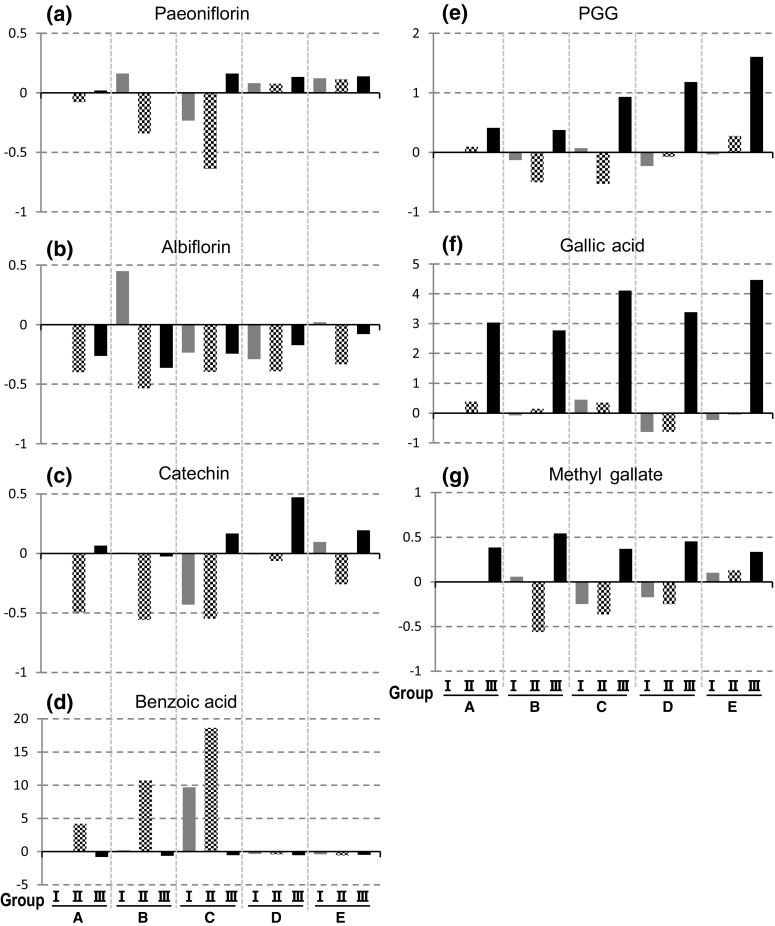
Changes in contents of the seven main components resulted from different processing methods, indicating as difference to the group A-I. Positive or negative value indicates increased- or decreased-folds of the content by reference to group A-I, respectively. **a** paeoniflorin, **b** albiflorin, **c** catechin, **d** benzoic acid, **e** PGG, **f** gallic acid, **g** methyl gallate

The harvested fresh roots of *P. lactiflora* were processed by 15 post-harvest processing methods, and then the eight main constituents in the produced roots were quantitatively analyzed to figure out the influence of different processing ways on the chemical composition of peony root. Five roots in each group treated by the same processing method were analyzed individually and the results are shown in Fig. 1s (supplementary data). Despite of individual differences in contents of the respective constituents, the five roots in the same group exhibited obviously similar chemical composition, reflecting as consistent trend of content changes among groups. This result clearly indicated that changes in chemical composition of the produced roots were undoubtedly caused by different processing methods. The average contents of the five individuals in each group are shown in Table 1 and Fig. 3. Paeonol was not detected in any of the roots. In addition, the changes in chemical composition resulted from the different processing methods could be clearly indicated by comparing contents of the respective components with those in the group A-I (Fig. 4).

**Table 1:** Contents of the seven constituents in peony root (mean ± S.D.,* n* = 5)

	Peoniflorin	Albiflorin	PGG	Catechin	Gallic acid	Methyl gallate	Benzoic acid
A-I	22.59 ± 1.35	4.63 ± 1.61	1.13 ± 0.38	0.97 ± 0.14	0.25 ± 0.07	0.23 ± 0.02	0.21 ± 0.06
A-II	20.81 ± 4.37	2.78 ± 1.43	1.24 ± 0.29	0.49 ± 0.26	0.35 ± 0.11	0.23 ± 0.02	1.13 ± 0.93
A-III	23.02 ± 1.47	3.42 ± 0.37	1.60 ± 0.07	1.03 ± 0.17	1.01 ± 0.10	0.31 ± 0.03	0.04 ± 0.01
B-I	26.26 ± 1.60	6.72 ± 2.80	0.99 ± 0.24	0.98 ± 0.58	0.23 ± 0.08	0.24 ± 0.02	0.25 ± 0.11
B-II	14.89 ± 3.48	2.16 ± 0.53	0.56 ± 0.24	0.43 ± 0.07	0.29 ± 0.11	0.15 ± 0.03	2.50 ± 0.93
B-III	22.72 ± 1.55	2.95 ± 0.58	1.56 ± 0.32	0.95 ± 0.17	0.94 ± 0.13	0.35 ± 0.03	0.08 ± 0.03
C-I	17.31 ± 6.04	3.54 ± 1.42	1.21 ± 0.27	0.55 ± 0.14	0.36 ± 0.07	0.17 ± 0.02	2.28 ± 1.06
C-II	8.14 ± 2.30	2.80 ± 2.16	0.52 ± 0.07	0.44 ± 0.13	0.34 ± 0.05	0.14 ± 0.03	4.19 ± 0.79
C-III	26.24 ± 4.03	3.50 ± 1.28	2.19 ± 0.26	1.13 ± 0.36	1.28 ± 0.11	0.32 ± 0.07	0.10 ± 0.02
D-I	24.42 ± 3.78	3.29 ± 0.90	0.87 ± 0.12	0.96 ± 0.22	0.09 ± 0.03	0.19 ± 0.02	0.14 ± 0.06
D-II	24.30 ± 2.51	2.82 ± 1.01	1.05 ± 0.16	0.91 ± 0.20	0.09 ± 0.03	0.18 ± 0.05	0.12 ± 0.04
D-III	25.61 ± 2.36	3.84 ± 1.54	2.48 ± 0.42	1.43 ± 0.32	1.10 ± 0.12	0.33 ± 0.04	0.10 ± 0.02
E-I	25.35 ± 1.86	4.73 ± 2.33	1.10 ± 0.37	1.06 ± 0.45	0.19 ± 0.09	0.25 ± 0.03	0.13 ± 0.05
E-II	25.16 ± 1.03	3.09 ± 1.10	1.45 ± 0.28	0.72 ± 0.22	0.24 ± 0.10	0.26 ± 0.04	0.09 ± 0.02
E-III	25.70 ± 3.13	4.27 ± 2.70	2.96 ± 0.22	1.16 ± 0.25	1.37 ± 0.13	0.31 ± 0.05	0.11 ± 0.02

As for the marker component paeoniflorin, the six groups treated with long-term low-temperature storage (D- and E-I–III) revealed very stable and high content (24.30–25.70 mg/g), whereas, the nine groups not stored at low temperature (A-, B- and C-I–III) showed considerable variation in paeoniflorin content, ranging from the lowest 8.14 mg/g to the highest 26.26 mg/g (Table 1). It was noticeable that groups A-II, B-II and C-II (roots were peeled after washing) and group C-I (roots were dried by drying machine but unpeeled) had a low content of paeoniflorin (8.14–20.81 mg/g), but significantly high content of benzoic acid (Fig. 3). The benzoic acid contents in the four groups (A-II, B-II, C-II and C-I) were even 4–18-fold higher than that in group A-I (Fig. 4d). From a viewpoint of chemical structures of these two components, such consistent changes suggested that the increment of benzoic acid resulted at least in part from degradation of paeoniflorin. Hayashi et al. [12] have reported that storing fresh roots under 20 °C for more than 22 days before further processing led to increase of paeoniflorin content and favorable whitish internal color. Our result was in good agreement with their finding. Compared with group A-I, paeoniflorin content in these six low-temperature storage groups increased slightly by around 10% (Fig. 4a). Long-term preservation at low-temperature after harvest might cause inactivation of a number of enzymes, including enzymes involved in paeoniflorin hydrolysis, therefore such processing prevented enzymatic decomposition and in consequent led to stable and high content of paeoniflorin in the low-temperature treated roots.

Of these six groups treated with low-temperature storage (Fig. 3), similar chemical compositions were observed between the respective group-pairs as D-I *vs.* E-I, D-II *vs.* E-II, and D-III *vs.* E-III, which were treated with the same handling process but dried under different drying conditions, indicating that long-term storing at low-temperature made the subsequent drying conditions, either indoor or by drying machine at 30 °C, less influence on the chemical compositions. In contrast, without the low-temperature storage after harvest, peeling (groups A-II, B-II, C-II) and hot-air drying (groups C-I, C-II) led to significant decrease in contents of various components except for benzoic acid.

Compared with the unpeeled roots in groups A-, B-, C-, D- and E-I, albiflorin content decreased obviously in the peeled roots in groups A-, B-, C-, D- and E-II–III (Figs. 3, 4b), even decreased more than 50% in group B-II (Fig. 4b). It has been reported that the periderm of peony root contains obviously higher amount of albiflorin than root body [13, 14]. In the present study, we also measured contents of the eight components in the periderm. Extremely high contents of albiflorin (13.78 mg/g) in the removed periderm was detected, which was approximately 3–5-fold higher than that in root body. These results revealed that the peeling process led to obvious decrease of albiflorin. The content of (+)-catechin (Fig. 4c) in the peeled roots in groups A-, B-, C-, D- and E-II decreased obviously as the same as the change of albiflorin, but increased in the roots which were both peeled and boiled, particularly in the groups C-, D- and E-III.

Obviously high contents of PGG, gallic acid and methyl gallate in the boiled roots (groups A-, B-, C-, D- and E-III) were observed (Fig. 3). Compared with the group A-I, the contents of these three components increased considerably in the five boiled groups (Fig. 4e–g). Investigation of the reasons responsible for such increment was further conducted as follows.

### Monitoring gallotannin changes by LC-ESI-IT-TOF–MS

**Fig. 5:**
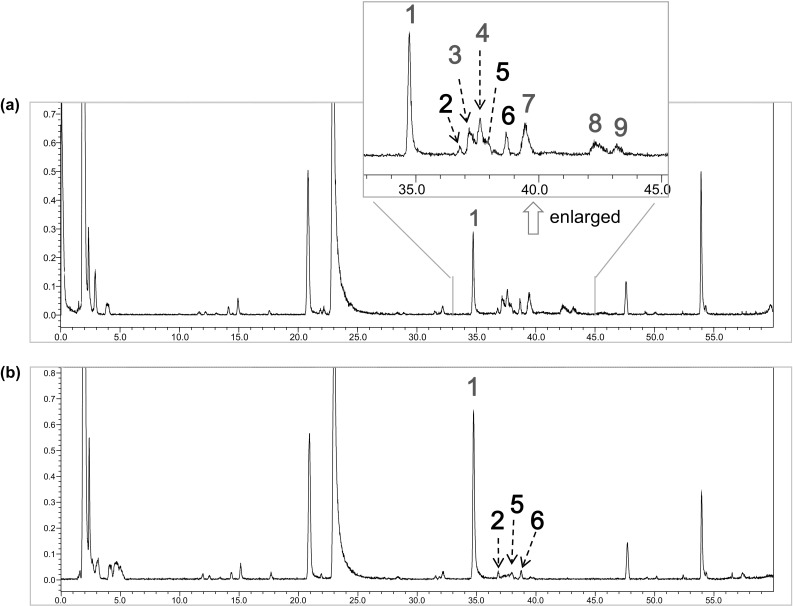
Total ion current **(**TIC) chromatograms of an unboiled root (**a**) and a boiled root (**b**). Identification of peaks 1–9 are indicated in Table 2

Compared with the unboiled roots, the contents of free gallic acid and PGG in the boiled root (groups A-, B-, C-, D-, E-III) significantly increased. Comparing the HPLC chromatographic profiles of these samples, it is conspicuous that a serials of peaks eluted out following PGG in the un-boiled roots became very low or disappeared in the boiled roots, whereas, the peaks of PGG and gallic acid increased obviously. Through LC–MS analysis (Fig. 5), these peaks were tentatively identified to be gallotannins as hexagalloyl- and heptagalloyl-glucoses by their stable [M–H]^−^ ions and MS/MS profiles (Table 2). The changes in peak area of these peaks are also indicated in Table 2. Gallotannins are a group of characteristic components in peony root, which are a subclass of hydrolysable tannins containing multiple galloyl units [3, 4, 15]. Nishizawa et al. [16, 17] have reported that polygalloylglucoses in peony root are exclusively based on a PGG core, and additional galloyl units are attached predominantly to the C-3 and C-6 positions in the glucose moiety through m- and p-depside linkages. The boiling process trigged decomposition of these gallotannins with 6–7 galloyl units in the roots of *P. lactiflora* to release high concentration of free gallic acid and PGG (Figs. 3, 4e, f). Especially extended boiling (120 min) resulted in complete degradation of the gallotannins having a high number of galloyl units [18]. Moreover, PGG has been reported to have diverse bioactivities, such as anti-allergic, anti-oxidative, anti-inflammatory effects. Gonzalez et al. [18] have reported that high concentration of the free gallic acid and PGG due to degradation of hydrolysable tannins after thermal process leads to obviously increase of the antioxidant ability of the witch hazel extracts. In the same manner, significant increase in contents of these components might give rise to enhanced antioxidant ability of the boiled roots, which is expected to strengthen its therapeutic efficacy.

**Table 2:** Identification of galloyltannins in unboiled and boiled peony roots by LC-IT-TOF–MS

Peak no.	*t*_*R*_ (min)	Assigned identity	Molecular formula	UV *λ*max (nm)	[M-H]^-^ *m/z*			[M-2H] ^2-^m/z	MS^2^ data (measured from [M-H]^-^)	Peak area in an unboiled root	Peak area in a boiled root
					Mean measured mass (Da)	Theoretical extract mass (Da)	Mass accuracy (ppm)				
1	34.74	1,2,3,4,6-penta-*O*-galloyl-β-d-glucose	C_41_H_32_O_26_	215, 279	939.1122	939.1104	1.9	469.0533	787.1028, 769.0926, 617.0836, 447.0612	13,655,477	28,387,184
2	36.80	Galloylalbiflorin	C_30_H_32_O_15_	217, 275	631.1652	631.1663	− 1.7		525.1618, 479.1658, 357.1617, 327.0884	1,139,911	1,714,129
3	37.20	Hexagalloyl glucose	C_48_H_36_O_30_	215, 279	1091.1211	1091.1213	− 0.2	545.0523	939.1287, 769.0826, 617.0715, 465.0638, 313.0510	3,523,516	–
4	37.61	Hexagalloyl glucose isomer	C_48_H_36_O_30_	215, 279	1091.1191	1091.1213	− 2.0	545.0506	939.1142, 769.0856, 617.0724, 465.0639, 313.0535	3,598,408	–
5	37.96	3′,6′-di-*O*-galloylpaeoniflorin	C_37_H_36_O_19_	215, 270	783.1805	783.1773	4.1		631.0608, 509.1684, 465.3314, 169.0940	478,539	453,623
6	38.68	unknown			509.2265				463.2218, 427.6630, 402.8793, 353.8746	2,790,645	2,784,044
7	39.48	Hexagalloyl glucose isomer	C_48_H_36_O_30_	215, 279	1091.1177	1091.1213	− 3.3	545.0511	939.1287, 769.0856, 617.0724, 465.0648, 313.0541	8,341,866	–
8	42.47	Heptagalloyl glucose	C_55_H_30_O_34_	215, 279	1243.1316	1243.1323	− 0.6	621.0574	621.0574, 545.0617, 469.5561, 349.0564	2,748,800	–
9	43.17	Heptagalloyl glucose isomer	C_55_H_30_O_34_	215, 279	1243.1316	1243.1323	− 0.6	621.0674	621.0674, 545.0519, 469.0580	1,984,263	–

### Internal color of the produced peony root

**Fig. 6:**
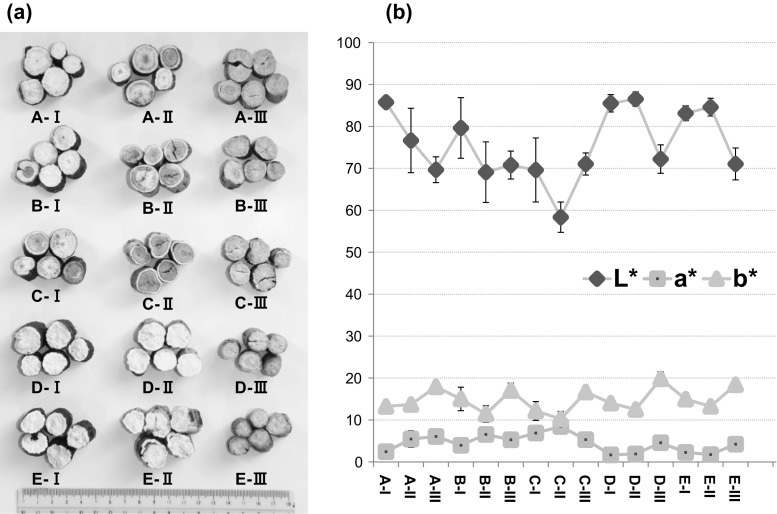
Photo of cross-section (**a**) and L*, a* and b* values measured by spectrophotometer (**b**) of the roots treated with 15 post-harvest processing methods

Morphological feature is also an important index to evaluate the processed herbal drugs. In the case of Chinese WPR and Japanese peony root, a plump root with whitish internal color is favored and traditionally believed to be of high quality. Therefore, the internal color of the dried roots was measured for the L*, a*, b* values by a spectrophotometer. Hayashi et al. [12] have reported that the L* value is an appropriate index to evaluate the degree of color change in cross-section of peony root, that is, the higher the L* value, the less the visible discoloration. Meanwhile, low absolute values of a* and b* also indicate less discoloration. The roots treated with long-term storage at low-temperature (groups D-I, D-II, E-I and E-II) showed the highest L* values (83.1–86.5), which were higher than the L* values of commercial peony root available in Japanese markets (data not shown). In contrast, the roots untreated with low-temperature storage showed varying degrees of discoloration. Particularly, internal color of the peeled roots in the groups A-II, B-II and C-II, as well as the unpeeled roots dried by drying machine at 30 °C in the group C-I turned to purple color, of which L* values were as low as 58.4–76.7. In addition, the boiled roots in the groups A-III, B-III, C-III, D-III and E-III showed similar L* values (69.7–72.2), despite the different processing methods, including low-temperature storage and different drying conditions. That was due to gelatinization of starch induced by the boiling process, thus resulting in pale yellow color of the whole internal part of root (Fig. 6).

**Fig. 7:**
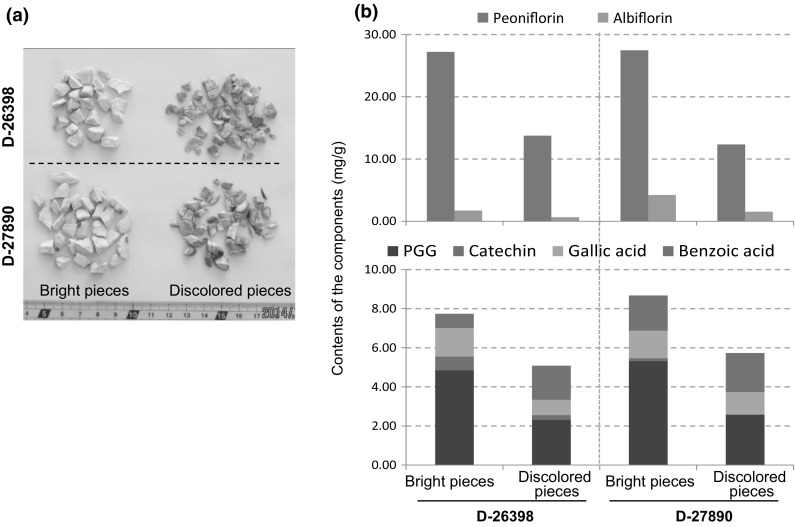
Photo of the bright pieces (left) and discolored pieces (right) included in the commercial samples of peony root, D-27890 and D-26398 (**a**) and contents of the six main components in these samples (**b**)

It is interesting to note that the discolored roots in groups A-II, B-II, C-II and C-I all had low contents of paeoniflorin and PGG, but high content of benzoic acid (Fig. 4a, d, e). It is not rare to find discolored pieces included in commercial samples of peony root. We supposed that those discolored pieces might also have low contents of paeoniflorin and PGG. To confirm this deduction, we picked up the discolored pieces from two commercial samples, and then quantitatively analyzed the eight main components in both bright and discolored pieces in the same sample, respectively. The results clearly indicated that the discolored pieces had obviously lower contents of paeoniflorin and PGG, but higher content of benzoic acid than the bright pieces (Fig. 7), which were in a good agreement with the deduction. This finding also provided underlying evidence supporting the traditional morphological feature-based assessment and suggested the whitish color is an important index related to the quality of peony root.

## Conclusion

The present study proposed 15 methods in view of the key steps involved in conventional post-harvest processing of peony root and applied these methods to the harvested fresh roots of a *P. lactiflora* cultivar. Through quantitative analysis of the eight main constituents in the produced roots and measurement of the internal root color, the impact of different processing methods, such as peeling, boiling, drying under different conditions, as well as long-term storage at low-temperature, on the chemical compositions and morphologic features of peony root were elucidated in detail. The low-temperature storage after harvest and the boiling process had synergistic promoting effect on increment of various components with bioactivity in peony root. Hot-air drying at 30 °C in a drying machine allowed fast drying, while also keeping the process of drying samples free from climate restriction. As a result, method E-III was preferred as a promising and practicable processing method for production of WPR and “Kiboshi Shakuyaku” to ensure high contents of the main active components in the processed root.

## Electronic supplementary material

Below is the link to the electronic supplementary material.

Supplementary material 1 (DOCX 80 kb)
