# Supplementary material for: Impact of different post-harvest processing methods on the chemical compositions of peony root
Source: J Nat Med. 2018 Apr 13;72(3):757–67. doi: 10.1007/s11418-018-1214-x (PMC6611895; doi:10.1007/s11418-018-1214-x)
Supplement: Supplementary file 1 — Supplementary material 1 (DOCX 80 kb) [file 11418_2018_1214_MOESM1_ESM.docx]

**Supplementary material**

**Fig. 1S** Contents of the 7 major components in individual root (No. 1-5) in each of the 15 groups treated by different post-harvest processing methods
